# On the Proportion of Graduates to the Population

**Published:** 1850-04

**Authors:** George Tucker

**Affiliations:** Formerly Professor of Moral Philosophy in the University of Virginia


### MEDICAL EXAMINER,

AND

### RECORD OF MEDICAL SCIENCE.

NEW SERIES.—NO. LXIV.—APRIL, 1850.

---

### ORIGINAL COMMUNICATIONS.

---

*On the proportion of Graduates to the Population.* By GEORGE TUCKER, Esq., formerly Professor of Moral Philosophy in the University of Virginia.

18 *Girard street, March* 16, 1850.

DEAR SIR:—My friend Professor Tucker, formerly Professor of Moral Philosophy and Political Economy in the University of Virginia,—whose attention has been much directed to statistical subjects, which he has, on many occasions, ably illustrated,—has sent me the accompanying communication, which confirms the view recently taken by my friend and colleague Dr. J. K. Mitchell, in his charge to the graduates of Jefferson Medical College—that the number of graduates annually sent from the schools is not yet sufficiently large to meet the demands of the community. Should you desire to publish it, it is at your service.

I am my dear sir,

Very truly yours,

ROBLEY DUNGLISON.

*Dr. F. G. Smith, Editor of the Medical Examiner.*

13 *Girard street, March* 16, 1850.

My Dear Sir:—I well recollect that when a committee of the Medical Association which met at Baltimore, in May, 1848, stated that the annual number of medical graduates exceeded the demands of the community, I remarked to you that I had drawn a contrary inference from the facts stated by the committee themselves; and your colleague Dr. Mitchell having, in his late charge to the graduates of Jefferson College, maintained, by a reference to statistical details, that the number annually added to the list of regular physicians was still far short of the wants of the country, I readily comply with your request, and state the grounds of my opinion on a subject on which learned doctors have thus differed. My estimate will be made for the year 1848.

On the first of June, of that year, the population of the United States—adding to the ordinary increase a reasonable allowance for the accessions from the acquired territories of Texas, California and New Mexico, and an unprecedented number of immigrants since 1840—was probably not less than 22,000,000. Let us, however, suppose it to be 21,500,000. With this population, what is the probable number of physicians? I have been in the habit of estimating one for every 800 persons. Dr. Mitchell, guided by the number in Philadelphia, supposes one for 700; but the proportion is always greater in the cities than in the country. Taking then the former ratio, the whole number of practising physicians in the United States is 26,875. On comparing the number of free white adults in the census of 1830, with that of 1840, and deducting from the latter the intervening gain from immigration, it would seem that the annual diminution by death in that class is about 2 per cent. For reasons with which I will not trouble you, I suppose the proportion of deaths in your profession to be greater than the average. Let us, however, assume it to be the same, and the annual reduction from this cause will be 537.

But the annual addition to the population requires a correspondent addition to the number of physicians. The yearly increase by natural multiplication appears to be about 2.8 per cent., which in a population of 21,500,000, amounted in 1848 to 602,000, to which 200,000 may be added for immigration. That year, indeed, the number of immigrants was considerably larger.

The whole addition to the population being thus 802,000, would consequently require 1002 physicians.

Further deductions must be made for those who quit the profession for other occupations, or because they are weary of it; for those who have obtained the degree of doctor of medicine but have never practiced; and for a small number who, having graduated in two schools, have been reckoned twice. I do not add to this list, those who practice in foreign countries, since the number of physicians who migrate to this country is probably equal to those who leave it. I have no data for estimating this class of deductions; but Dr. Mitchell reckons it at 2 per cent., and you think that estimate not too high. This would take off 537 more, or at 1 per cent. 268.

The additional number of physicians annually required to supply the wants of the country was then as follows:

| | |
|---|---|
| From deaths, | 537 |
| From increase of population, | 1002 |
| From withdrawals, &c., at 2 per cent., | 537 |
| | 2076 |

If, however, we reduce the last head to only 1 per cent., the number would be 1807, so that if the annual number of medical graduates had not exceeded 1500, as the report of that committee assumed, we find that after allowing the widest range for possible error, the annual supply is from 20 to 35 per cent. less than the annual demand. This deficiency, which has since increased, was of course supplied from the large class of uneducated and half-educated practitioners who are still suffered to sport with the health and lives of the credulous multitude.

With great respect and regard, I am, &c.,

George Tucker.

*To Dr. R. Dunglison.*

